# Altered Oxygen Utilisation in Rat Left Ventricle and Soleus after 14 Days, but Not 2 Days, of Environmental Hypoxia

**DOI:** 10.1371/journal.pone.0138564

**Published:** 2015-09-21

**Authors:** James A. Horscroft, Sarah L. Burgess, Yaqi Hu, Andrew J. Murray

**Affiliations:** Department of Physiology, Development & Neuroscience, University of Cambridge, Downing Street, Cambridge, CB2 3EG, United Kingdom; Hull York Medical School, UNITED KINGDOM

## Abstract

The effects of environmental hypoxia on cardiac and skeletal muscle metabolism are dependent on the duration and severity of hypoxic exposure, though factors which dictate the nature of the metabolic response to hypoxia are poorly understood. We therefore set out to investigate the time-dependence of metabolic acclimatisation to hypoxia in rat cardiac and skeletal muscle. Rats were housed under normoxic conditions, or exposed to short-term (2 d) or sustained (14 d) hypoxia (10% O_2_), after which samples were obtained from the left ventricle of the heart and the soleus for assessment of metabolic regulation and mitochondrial function. Mass-corrected maximal oxidative phosphorylation was 20% lower in the left ventricle following sustained but not short-term hypoxia, though no change was observed in the soleus. After sustained hypoxia, the ratio of octanoyl carnitine- to pyruvate- supported respiration was 11% and 12% lower in the left ventricle and soleus, respectively, whilst hexokinase activity increased by 33% and 2.1-fold in these tissues. mRNA levels of PPARα targets fell after sustained hypoxia in both tissues, but those of PPARα remained unchanged. Despite decreased *Ucp3* expression after short-term hypoxia, UCP3 protein levels and mitochondrial coupling remained unchanged. Protein carbonylation was 40% higher after short-term but not sustained hypoxic exposure in the left ventricle, but was unchanged in the soleus at both timepoints. Our findings therefore demonstrate that 14 days, but not 2 days, of hypoxia induces a loss of oxidative capacity in the left ventricle but not the soleus, and a substrate switch away from fatty acid oxidation in both tissues.

## Introduction

The partial pressure of atmospheric O_2_ (*P*_O2_) is the driving force behind O_2_ delivery to respiring tissues, and thus O_2_ delivery may be compromised when atmospheric *P*_O2_ is low. Mitochondria represent the primary sites of O_2_ consumption in most cells of the body, as O_2_ accepts electrons at complex IV of the electron transport chain, allowing the oxidation of substrates–a process coupled to the synthesis of ATP. Under hypoxic conditions, O_2_ can instead accept a single electron at complex III, resulting in the production of superoxide (O_2_^.-^), which can subsequently yield other reactive oxygen species (ROS) [1]. Although ROS act as signalling molecules in low concentrations, pathologically high levels of ROS can impair mitochondrial or cellular function by damaging proteins, lipids and DNA [2]. Thus, alterations in mitochondrial function may represent a path towards acclimatisation under hypoxic conditions [3], potentially altering ROS production and susceptibility to oxidative stress.

Mechanisms underlying metabolic acclimatisation frequently result from stabilisation of the hypoxia inducible factor (HIF) transcription factors, with HIF-1 thought to mediate the short-term response to hypoxia but HIF-2 stabilisation underlying the response to more sustained hypoxia [4]. HIF-stabilisation may lead to an attenuation of oxidative phosphorylation; indeed oxidative metabolism was augmented in the gastrocnemius muscle of mice in which skeletal muscle HIF-1α had been selectively deleted [5]. This may be a consequence of a HIF interaction with peroxisome proliferator-activated receptor-gamma coactivator 1-alpha (PGC-1α) [6] and BCL2/adenovirus E1B 19 kDa interacting protein 3 (BNIP3) [7], which regulate mitochondrial biogenesis and autophagy, respectively. Moreover, in some tissues, HIF-1α may suppress transcription of the targets of peroxisome proliferator-activated receptor 1-alpha (PPARα), a transcription factor which regulates expression of genes involved in the β-oxidation of fatty acids, potentially through decreasing PPARα expression [8] or its DNA binding activity [9].

In humans, both 20 h in a normobaric hypoxia chamber [10] and sustained exposure to hypobaric hypoxia at high altitude [11] resulted in impaired cardiac energetics, measured as phosphocreatine-to-ATP ratio using ^31^P-NMR spectroscopy, although the underlying mechanisms were probably not the same in these two cases. The response to sustained hypoxia may be a consequence of a decrease in respiratory capacity, which has been reported in isolated mitochondria [12] and permeabilised fibres [13] from the hypoxic rat heart—an effect which can be prevented by supplementation with dietary nitrate [13]. Moreover, sustained hypoxia has been shown to decrease expression of PPARα target genes such as carnitine palmitoyl-transferase 1 (CPT1) and uncoupling protein 3 (UCP3), and increase expression of genes associated with glucose metabolism in the left [14] and right [15] ventricles of the rat heart. This could underlie a substrate switch away from fatty acid oxidation towards more O_2_-efficient carbohydrate metabolism [16], although notably no such change was observed in the mouse heart after 7 d hypoxic exposure [17]. These changes were associated with decreased respiratory capacity and rate of ATP synthesis, yet mitochondrial coupling was preserved, despite the changes in expression of UCP3 [14].

In skeletal muscle acclimatising to hypoxia, alterations in mitochondrial function seem to occur independently of alterations in mitochondrial volume density [18]. At high altitude, the respiratory capacity of human skeletal muscle was unchanged after 9–11 d hypoxia [19], but lowered after 28 d at a lower altitude with no discernible difference in mitochondrial volume density [20]. With sustained exposure to extreme high altitude a loss of muscle mitochondrial density does occur [21,22], though it is unclear whether this occurs as a consequence of the greater degree of hypoxia, the longer duration of exposure or another complication of the extreme high altitude environment (e.g. a detraining effect). A substrate switch does appear to occur in hypoxic skeletal muscle, as 21 d at altitude lowered rates of fatty acid oxidation capacity [23] and increased rates of carbohydrate consumption [24], potentially as a consequence of decreased PPARα signalling. UCP3 expression decreases in human skeletal muscle after extreme high altitude [21], but whether this is associated with a change in coupling remains to be established.

We hypothesised that 14 days environmental hypoxia would alter mitochondrial respiratory function and substrate preference in rat heart and skeletal muscle, but that this would be unaltered after 2 days of exposure. In addition, we hypothesised that UCP3 expression would fall in these tissues following 14 days hypoxic exposure, and that this would be associated with an increase in mitochondrial coupling.

To investigate this, we exposed rats to normoxia (21% O_2_), or environmental hypoxia (10% O_2_) for 2 or 14 days. Mitochondrial respiratory function was assessed in cardiac left ventricle and soleus muscle using high-resolution respirometry. Since a loss of respiratory capacity could be due to changes in mitochondrial density, or qualitative changes in mitochondrial function, the resulting respiration rates were normalised both to the mass of muscle tissue studied and activity of the TCA cycle enzyme, citrate synthase in the sample. To investigate putative mechanisms, expression of PPARα and some of its targets were measured, alongside the activity of metabolic enzymes. Additionally, UCP3 levels were measured by immunoblotting and analysis of protein carbonyls was performed as an indicator of oxidative stress.

## Materials and Methods

### Ethical approval

All experiments were carried out by a personal licence holder, conformed to UK Home Office guidelines under the Animals in Scientific Procedures Act and were reviewed by the University of Cambridge Animal Welfare and Ethical Review Committee.

### Animals

**Fig 1:**
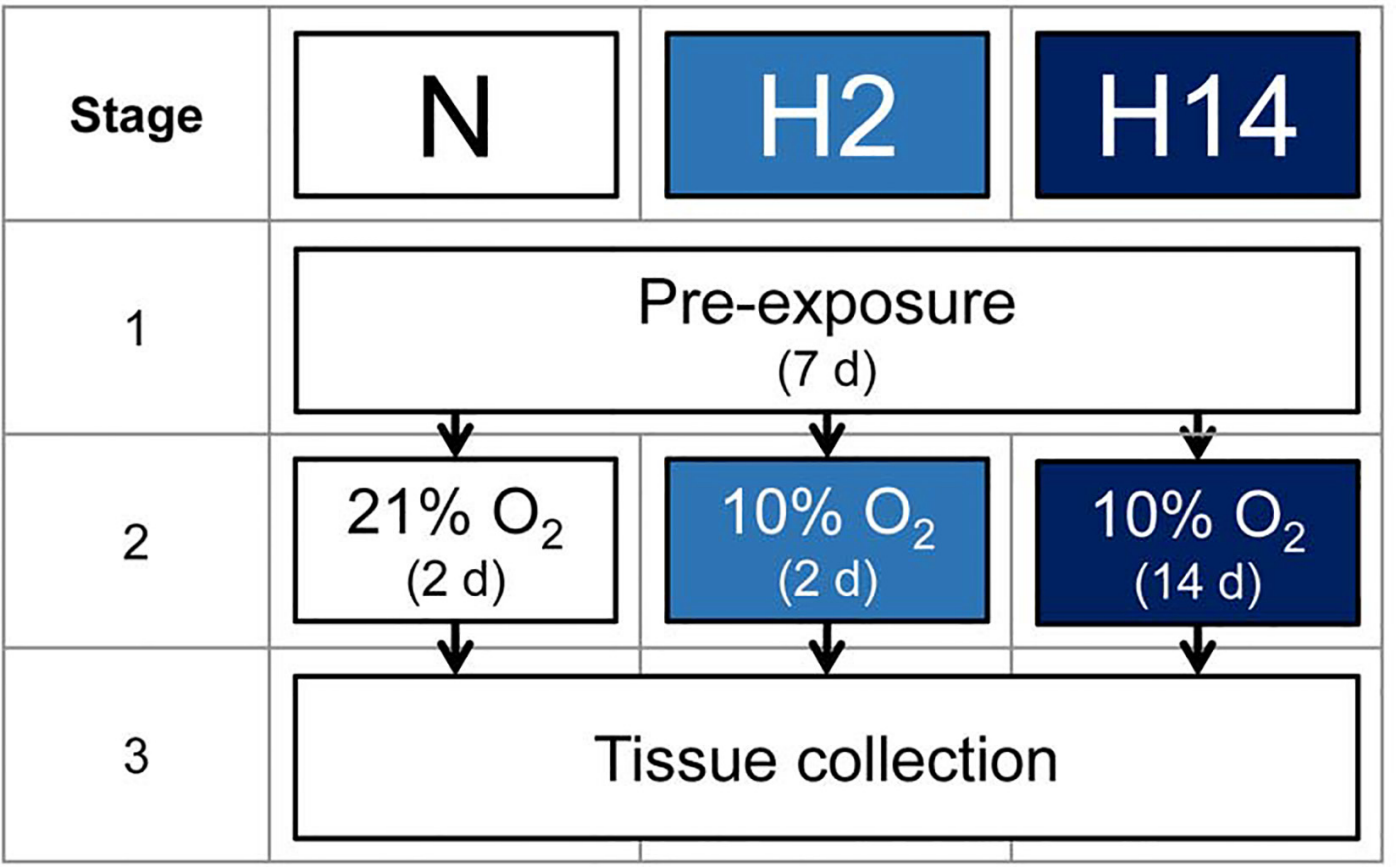
Study design. Rats were assigned to three groups: normoxia (N), 21% atmospheric O_2_ for 2 d; short-term hypoxia (H2), 10% atmospheric O_2_ for 2 d; or sustained hypoxia (H14), 10% atmospheric O_2_ for 14 d.

Male Wistar rats (*n* = 21) were purchased from Charles River (Scientific Products Farm Ltd., UK) and were single-housed in a temperature- (21°C), humidity- (46%) and light-controlled (12 h/12 h light/dark cycle) environment with a standard diet (RMIP, Special Diets Services, UK) and distilled water provided *ad libitum*. After 7 days, rats were either kept under normoxic conditions (21% O_2_) for a further 2 days (normoxia, *n* = 7), or transferred to a hypoxia chamber (PFI Systems Ltd., Milton Keynes, UK) maintained at 10% O_2_ with 20 air changes/hour for 2 days (short-term hypoxia, *n* = 7) or 14 days (sustained hypoxia, *n* = 7) (Fig 1). Body mass, food intake and water intake were recorded daily.

Rats were anaesthetised by subcutaneous injection of 25% Hypnorm (Vetapharma), 50% distilled water and 25% midazolam (Hypnovel, Roche) at a dosage of 2 ml kg^-1^ body mass. After cessation of peripheral sensitivity, the chest cavity was opened and blood collected from the left ventricle by cardiac puncture. A droplet of blood was loaded into a microcuvette for quantification of haemoglobin concentration using a HemoCue Hb 201 Analyzer (Ängleholm, Sweden). The heart was excised and weighed and a portion of the left ventricle, as well as the soleus muscle, was placed in ice-cold biopsy preservation medium (BIOPS: 2.77 mM CaK_2_EGTA, 7.23 mM K_2_EGTA, 6.56 mM MgCl_2_.6H_2_O, 20 mM taurine, 15 mM phosphocreatine, 20 mM imidazole, 0.5 mM dithiothreitol, 50 mM MES, 5.77 mM Na_2_ATP, pH 7.1) for analysis by high-resolution respirometry, while another portion of the left ventricle and the other soleus were snap-frozen.

### High-resolution respirometry

Muscle fibres from left ventricle and soleus were dissected and permeabilised as described previously [25,26]. Briefly, the tissues were dissected into fibre bundles and incubated for 20 min at 4°C with gentle rocking in BIOPS with 72 μg μl^-1^ saponin to selectively permeabilise the plasma membrane, leaving mitochondrial membranes intact. Fibres were then washed three times for 5 min at 4°C with gentle rocking in respiration medium (MiR05: 0.5 mM, EGTA, 3 mM MgCl_2_.6H_2_O, 60 mM K-lactobionate, 20 mM taurine, 10 mM KH_2_PO_4_, 20 mM HEPES, 110 mM sucrose, 1 g L^-1^ defatted BSA, pH 7.4) [26].

Cardiac (2–3 mg) or soleus (4–6 mg) fibre bundles were added to Oxygraph-O2k (Oroboros Instruments, Innsbruck, Austria) chambers containing 2 ml MiR05 at 37°C, and the titration performed was based on those described previously [20,26]. Normal Leak respiration (L_N_) was stimulated through addition of malate (2 mM) and octanoyl-carnitine (0.2 mM). ADP (5 mM) was added to trigger oxidative phosphorylation limited by β-oxidation (P_β_) [20,26]. Following this pyruvate (20 mM) was added to quantify oxidative phosphorylation associated with TCA cycle flux (P_P+β_) [20,26]. Maximisation of electron flux through complex I and complex II was achieved through addition of glutamate (10 mM, P_I+β_) and succinate (10 mM, P_I+II+β_), respectively [20,26]. Cytochrome *c* (10 μM) was then used to test the integrity of the outer membrane, before rotenone (0.5 μM) was added to inhibit complex I, resulting in a rate of oxidative phosphorylation limited by complex II (P_II+β_) [20,26].

Oxygen concentration in the chambers was maintained at 250 to 400 μM by periodic oxygenation in order to negate limitations of oxygen diffusion. All respiration rates were corrected for the background oxygen flux in the absence of tissue and were subsequently normalised to (i) tissue wet mass and/or (ii) citrate synthase activity. Respiratory chamber homogenates were prepared for the citrate synthase activity assay as previously described [20]. The increase in respiration rate induced by ADP was expressed as a ratio of total respiration rate after ADP addition to give the oxidative phosphorylation coupling efficiency (OCE):

$$\text{OCE}=\frac{{\text{P}}_{\beta }-{\text{L}}_{\text{N}}}{{\text{P}}_{\beta }}$$

The ratio of octanoyl carnitine- to pyruvate-supported oxidative phosphorylation was also calculate to indicate preference for fatty acid oxidation (FAO).

### Enzyme activity assays

Approximately 10 mg of whole left ventricle or soleus was crushed with a pestle and mortar pre-cooled with liquid nitrogen. The tissue was then placed in 300 μl homogenisation buffer (100 mM KH_2_PO_4_, 5 mM EDTA, 0.1% v/v Triton X-100) and homogenised with 20 plunges at 1,200 rpm on a Potter-Elvejhem homogeniser (Velp Scientifica, Italy) and 30 s on a Polytron (PT 10–35 GT, Kinematica Inc., Switzerland). Samples were then centrifuged (200 × *g*, 30 s, 4°C), the supernatants collected and stored at -80°C until use. Protein concentration of chamber and tissue homogenates was measured using the Quick Start Bradford protein assay (Bio Rad).

Citrate synthase activity of chamber and tissue homogenates was quantified at 25°C as described previously [27]. The assay buffer contained 20 mM Tris, 0.1 mM 5,5’-dithiobis-2-nitrobenzoic acid and 0.3 mM of acetyl CoA at pH 8.00. The reaction were initiated by the addition of 0.5 mM oxaloacetate and absorbance change at 412 nm was measured.

3-hydroxy acyl dehydrogenase (HADH) activity was assayed at 30°C as described previously [21]. The assay buffer contained 50 mM imidazole, 0.15 mM NADH and 0.1% v/v Triton X-100 at pH 7.40. The reaction was initiated by the addition of 0.1 mM acetoacetyl CoA and absorbance change at 340 nm was measured.

Hexokinase activity was quantified at 30°C in an assay buffer comprising 20 mM imidazole, 1 mM ATP, 5 mM 7H_2_O.MgCl_2_, 5 mM dithiothreitol, 2 mM NAD^+^, and 3.125 U glucose-6-phosphate-dehydrogenase (G6PDH) at pH 7.40. 5 mM glucose was added to trigger the reaction and absorbance change at 340 nm was measured.

Activity of lactate dehydrogenase (LDH) was quantified at 30°C essentially as described previously [28]. The assay buffer contained 50 mM HEPES and 0.3 mM NADH at pH 7.00 and the reaction was triggered by the addition of 0.5 mM pyruvate. The reaction was monitored by measuring absorbance at a wavelength of 340 nm.

### Reverse transcription and RT-PCR

RNA was extracted from frozen left ventricle and soleus using a Qiagen RNeasy Fibrous Tissue Mini kit according to manufacturer’s instructions. 5 μM random hexamers and 1 mM deoxynucleotide triphosphates were combined with 1 μg RNA in a 10 μl solution. Primer annealing was initiated by incubation at 65°C for 5 min in a Veriti 96-well Thermocycler (Applied Biosystems). Samples were combined with 200 U SuperScript III reverse transcriptase, 40 U RNaseOUT recombinant RNase inhibitor, 200 mmol dithiothreitol, 100 mmol MgCl_2_ and 1 × reverse transcriptase buffer (Life Technologies) in a reaction volume of 20 μl. Following initial incubation in the Thermocycler (10 min, 25°C), complementary DNA was synthesised (50 min, 50°C), after which the reaction was terminated (5 min, 85°C). Real-time quantitative PCR was performed in triplicates in 96-well plates on a StepOne Plus detection system (Applied Biosciences) with an initial incubation period (10 min at 95°C), then 40 cycles of elongation (15 s at 95°C) and cooling (1 min at 60°C). Taqman probe/primer assay mix (Life Technologies) for *Ppara*, *Ucp3*, *Acadm* and *Cpt1b* were used in Taqman Universal PCR Master Mix. Expression levels of targets were normalised to *Hprt* by the ΔCT method.

### Immunoblotting

SDS-PAGE and immunoblotting were used to measure protein levels in heart and soleus homogenates, as described previously [13]. The quality of the transfer was checked with Ponceau staining and homogeneity between gels ensured by loading normoxic control samples to each gel. Bands were quantified using UN-SCAN-It software (Silk Scientific, Orem, UT, USA). UCP3 protein levels were detected using an antibody purchased from Abcam (Cambridge, UK, ab3477). The Oxyblot Protein Oxidation Detection Kit (Merck Millipore, UK) was used as described in the manufacturer’s instructions to measure protein carbonyls. Modification of proteins by ROS and other free radical species was quantified following derivatisation of the carbonyl groups with 2,4-dinitrophenylhydrazine. SDS-PAGE and immunoblotting were then performed with a primary antibody against the derivatised carbonyl groups.

### Statistics

For comparisons between normoxia, short-term hypoxia and sustained hypoxia groups, a one-way ANOVA was performed. Where significant differences were found, *post-hoc* pairwise comparisons were carried out with a Tukey correction. All analyses were carried out using GraphPad Prism 6 software (GraphPad Software, Inc.) and differences were considered significant when *p* ≤ 0.05. Data are expressed as mean ± standard error of the mean (SEM). All data are available in the Supporting Information file (S1 Dataset).

## Results

### Morphology

**Fig 2:**
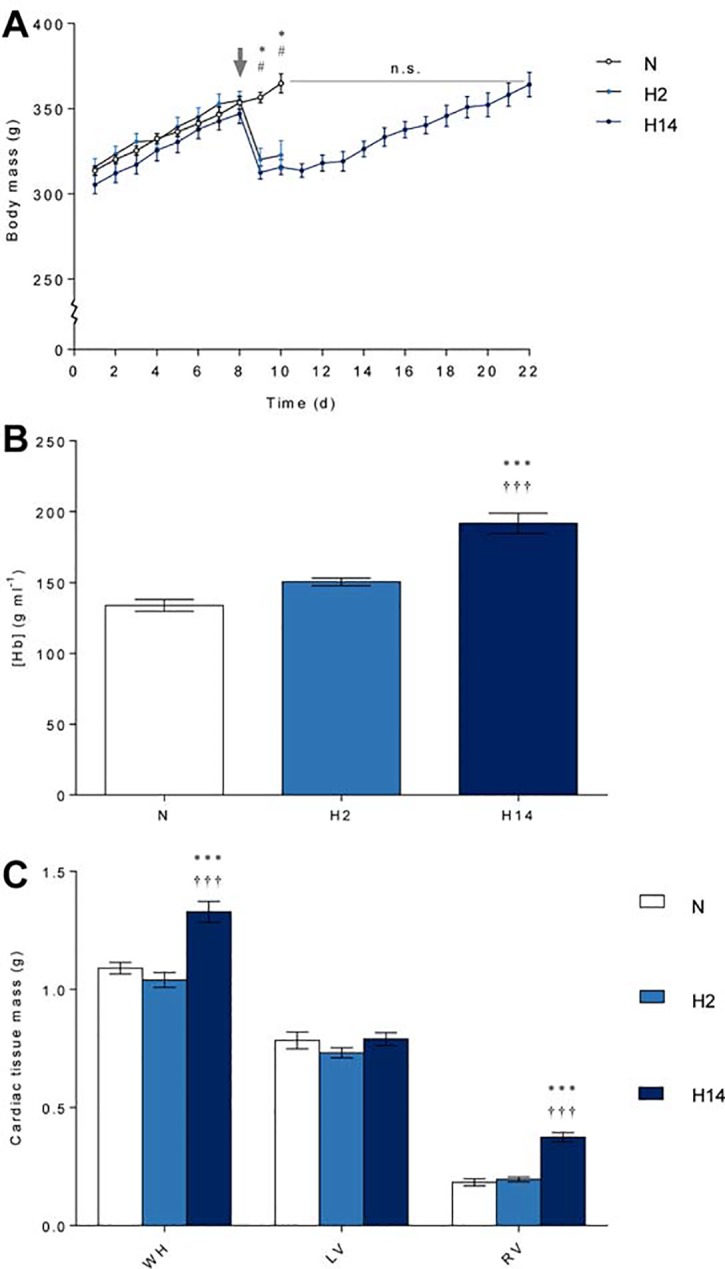
Morphology. Daily body mass (A), end haemoglobin concentration ([Hb]) (B), and end mass of whole heart (WH), left ventricle (LV) and right ventricle (RV) (C) after normoxia (N, white), short-term hypoxia (H2, light blue) or sustained hypoxia (H14, dark blue). * *p* ≤ 0.05, *** *p* ≤ 0.001 for sustained hypoxia vs. normoxia; # *p* ≤ 0.05 for short-term hypoxia vs. normoxia; ††† *p* ≤ 0.001 for sustained vs. short-term hypoxia. Data represent mean ± SEM.

Body mass did not differ between groups during the pre-exposure period. Final body mass of the short-term hypoxia (10% O_2_) group was 12% (*p* ≤ 0.001) lower than that of normoxic controls (Fig 2A). However, final body mass of the sustained hypoxia group did not differ to normoxic controls (Fig 2A), due to an initial loss of weight, which recovered over the subsequent 12 days. Daily food and water intake did not differ between groups during the 7 d pre-exposure period (Table 1). However, during hypoxic exposure, average daily food intake was 63% (*p* ≤ 0.001) and 28% (*p* ≤ 0.001) lower in the short-term and sustained hypoxia groups, respectively, relative to normoxic controls (Table 1). Daily water intake was also 42% (*p* ≤ 0.001) lower in the short-term hypoxia group, although in the sustained hypoxia group this did not differ from normoxic controls (Table 1).

**Table 1:** Food and water intake. Rats were housed in normoxia for 7 d (pre-exposure stage), then normoxia for 2 d, hypoxia (10% O_2_) for 2 d, or hypoxia (10% O_2_) for 14 d (exposure stage).

	STAGE	Normoxia	2 d hypoxia	14 d hypoxia
**Food intake**(mg kg^-1^ d^-1^)	Pre-exposure	94 ± 2	94 ± 2	97 ± 1
	Exposure	86 ± 3	32 ± 7 ***	62 ± 1 ** †††
**Water intake** (ml kg^-1^ d^-1^)	Pre-exposure	110 ± 6	120 ± 7	113 ± 6
	Exposure	102 ± 5	59 ± 9 ***	87 ± 5 †

** p **≤** 0.01

*** p **≤** 0.001 *vs*. normoxia

† *p* **≤** 0.05

††† *p* **≤** 0.001 *vs*. 2 d hypoxia.

Sustained hypoxia resulted in a 43% (*p* ≤ 0.001) increase in haemoglobin concentration ([Hb]), but there was no change with short-term hypoxia (Fig 2B). Whole heart mass was 22% (*p* ≤ 0.001) higher after sustained but not short-term hypoxia compared to normoxia (Fig 2C). This seemed to be a consequence of ventricular remodelling as left ventricle mass was similar between all groups, whilst right ventricle mass was 2.1-fold (*p* ≤ 0.001) higher after sustained but not short-term hypoxia relative to normoxia (Fig 2C).

### Left ventricle mitochondrial function

**Fig 3:**
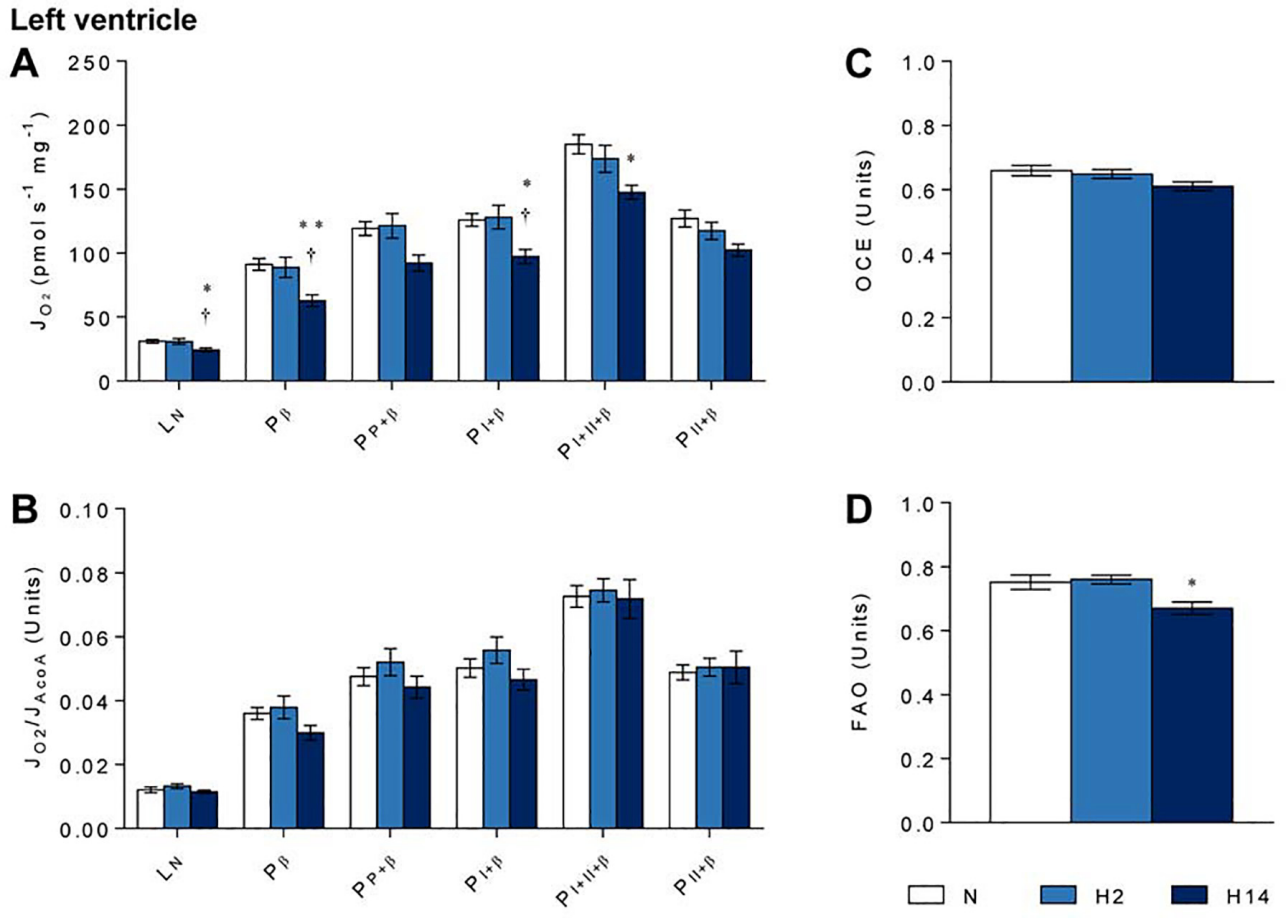
Left ventricle mitochondrial function. Respiration rates corrected for wet mass (A) and citrate synthase activity (B); oxidative phosphorylation coupling efficiency (OCE) as a marker of mitochondrial coupling (C); and ratio between octanoyl carnitine- and pyruvate-supported respiration (FAO) as a marker of preference for fatty acid oxidation (D) after normoxia (N, white), short-term hypoxia (H2, light blue) or sustained hypoxia (H14, dark blue). * *p* ≤ 0.05, ** *p* ≤ 0.01 vs. normoxia; † *p* ≤ 0.05 vs. short-term hypoxia. Data represent mean ± SEM.

In the left ventricle of the heart, short-term hypoxia had no effect on any measure of mass-specific mitochondrial function (Fig 3A). With sustained hypoxia, however, mass-corrected leak respiration (L_N_) and octanoyl carnitine-supported oxidative phosphorylation (P_β_) were 21% (*p* ≤ 0.05) and 31% (*p* ≤ 0.01) lower than in normoxic controls (Fig 3A), respectively. After activation of the TCA cycle (P_P+β_), no statistically significant differences between groups were observed, though there was a near-significant trend towards a decrease in P_P+β_ following sustained hypoxia (*p* = 0.073). Mass-corrected complex I-supported oxidative phosphorylation (P_I+β_) was lowered by 23% (*p* ≤ 0.05) due to sustained hypoxia, while complex II-supported oxidative phosphorylation (P_II+β_) was not affected (Fig 3A). Mass-corrected maximal oxidative phosphorylation (P_I+II+β_) was 20% (*p* ≤ 0.05) lower after sustained hypoxia relative to normoxia (Fig 3A).

Despite these differences in mass-corrected respiration, when corrected to citrate synthase activity no respiration rates appeared to be changed by either duration of hypoxic exposure (Fig 3B). Mitochondrial coupling (OCE) was unaffected by both short-term and sustained hypoxia (Fig 3C), though preference for FAO was 11% (*p* ≤ 0.05) lower after sustained hypoxia relative to normoxia (Fig 3D), suggesting a substrate switch away from fatty acids in the heart.

### Regulation of metabolism in the left ventricle

**Fig 4:**
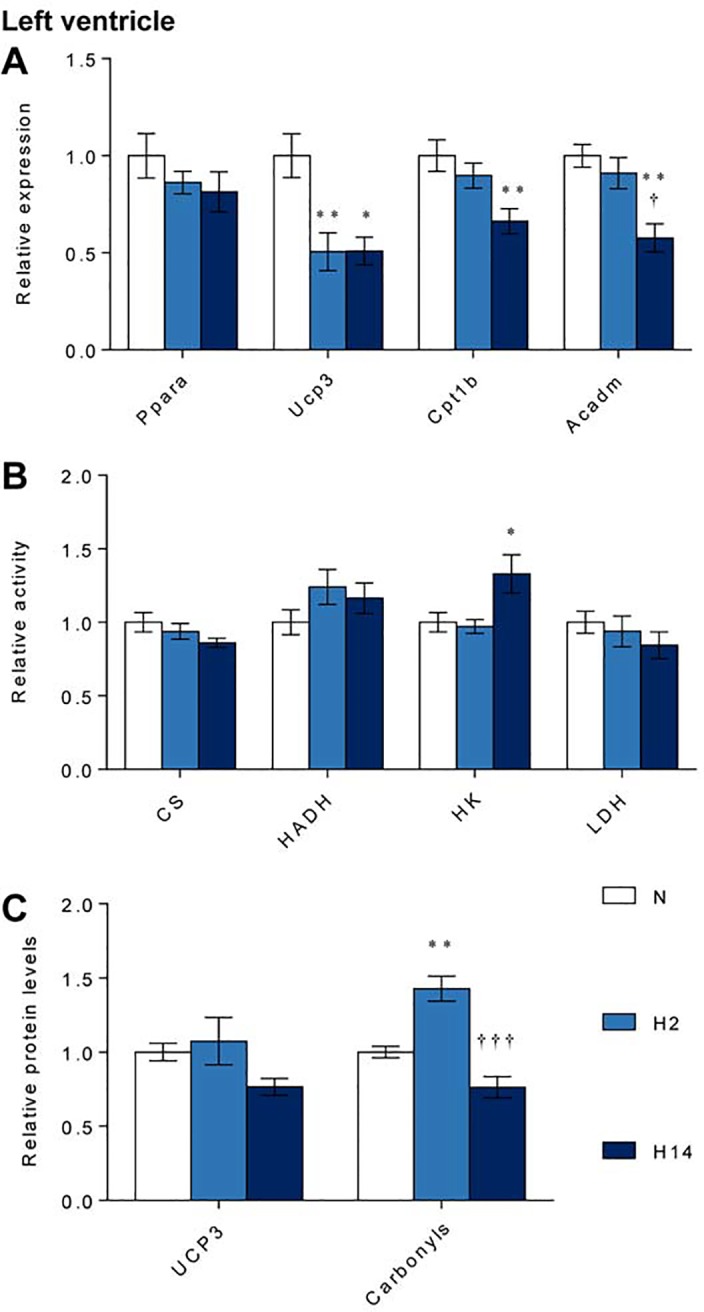
Regulation of metabolism in the left ventricle. mRNA levels of *Ppara* and its targets (A); activity of citrate synthase (CS), 3-hydroxyacyl dehydrogenase (HADH), hexokinase (HK) and lactate dehydrogenase (LDH) (B); and levels of UCP3 and protein carbonyls (C) after normoxia (N, white), short-term hypoxia (H2, light blue) or sustained hypoxia (H14, dark blue). * *p* ≤ 0.05, ** *p* ≤ 0.01 *vs*. normoxia; † *p* ≤ 0.05 *vs*. short-term hypoxia. Data represent mean ± SEM.

Expression of *Ppara* in the left ventricle of the heart was unaffected by either duration of hypoxic exposure (Fig 4A). However, hypoxia decreased expression of three targets of PPARα, *Ucp3*, *Cpt1b* and *Acadm*, suggesting that PPARα transcriptional activity is regulated during hypoxic exposure. In comparison with normoxic controls, *Ucp3* expression was 50% (*p* ≤ 0.01) lower after short-term hypoxia and 49% (*p* ≤ 0.05) lower after sustained hypoxia (Fig 4A). Short-term hypoxia was insufficient to induce changes in expression of *Cpt1b* or *Acadm*, though with sustained hypoxia expression of these genes were 34% (*p* ≤ 0.01) and 37% (*p* ≤ 0.01) lower, respectively (Fig 4A).

Activity of citrate synthase (CS), 3-hydroxyacyl dehydrogenase (HADH) and lactate dehydrogenase (LDH) in whole left ventricle were unaltered by short-term or sustained hypoxia (Fig 4B). Hexokinase (HK) activity was 33% (*p* ≤ 0.05) higher after sustained hypoxia, relative to normoxic controls (Fig 4B), which is indicative of an increase in glycolytic capacity.

Despite the changes in *Ucp3* expression, protein levels of UCP3 were not significantly different between hypoxic groups and normoxic controls, though UCP3 protein levels were lower at 14 days than after 2 days hypoxia (*p* ≤ 0.05) (Fig 4C). Protein carbonyl levels were 40% (*p* ≤ 0.01) higher after short-term hypoxic exposure, but returned to normoxic levels after sustained hypoxia (Fig 4C), suggesting a transient increase in oxidative stress.

### Soleus mitochondrial function

**Fig 5:**
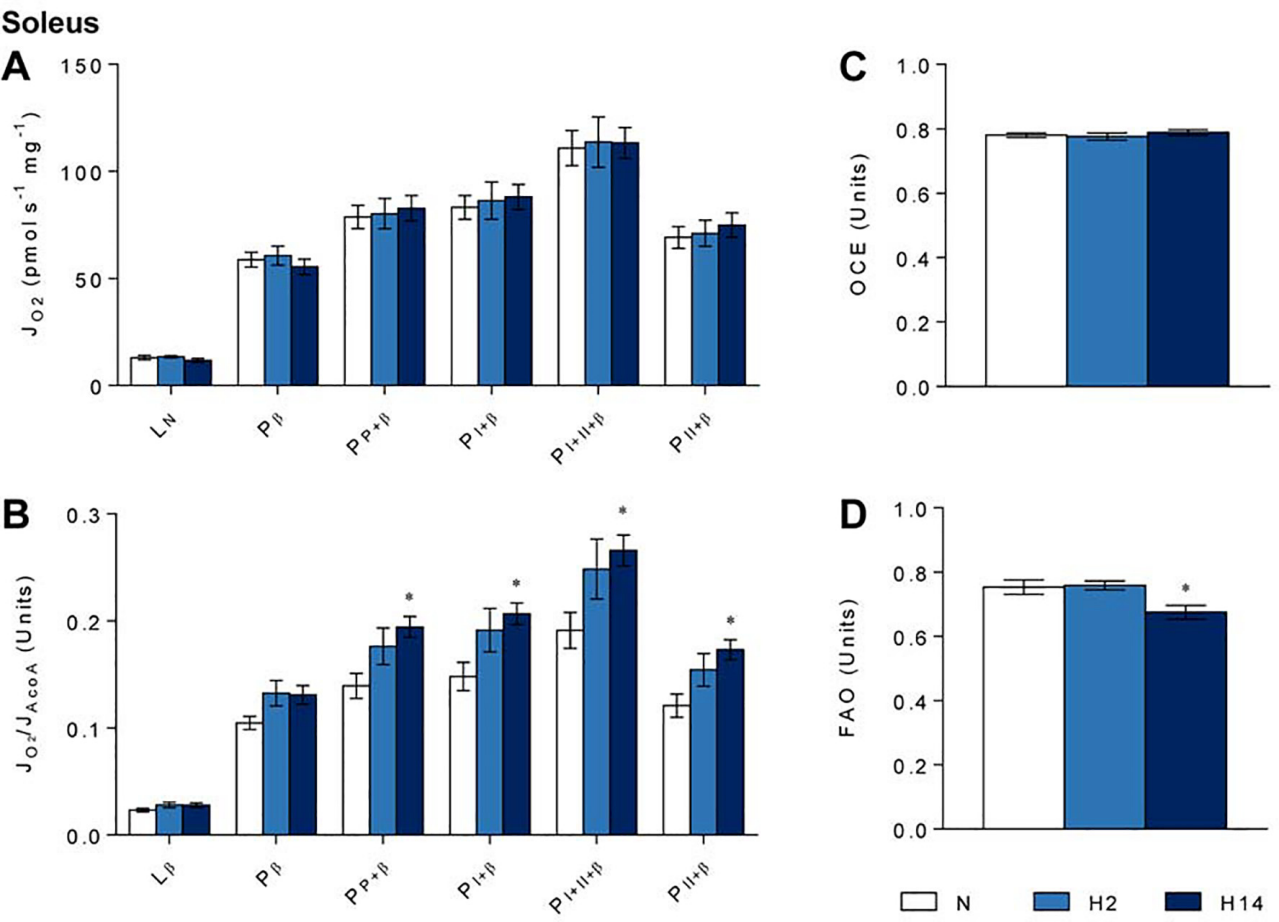
Soleus mitochondrial function. Respiration rates corrected for wet mass (A) and citrate synthase activity (B); oxidative phosphorylation coupling efficiency (OCE) as a marker of mitochondrial coupling (C); and ratio between octanoyl carnitine- and pyruvate-supported respiration (FAO) as a marker of preference for fatty acid oxidation (D) after normoxia (N, white), short-term hypoxia (H2, light blue) or sustained hypoxia (H14, dark blue). * *p* ≤ 0.05, ** *p* ≤ 0.01 *vs*. normoxia; † *p* ≤ 0.05 *vs*. short-term hypoxia. Data represent mean ± SEM.

In contrast with the left ventricle of the heart, all measures of mass-corrected respiration were unaffected by either short-term or sustained hypoxia in soleus muscle (Fig 5A). Citrate synthase-corrected oxidative phosphorylation, however, was increased in the skeletal muscle. Pyruvate-supported oxidative phosphorylation (P_P+β_) was 36% (*p* ≤ 0.05) higher after sustained hypoxia relative to normoxic controls, while octanoyl carnitine-supported oxidative phosphorylation (P_β_) remained unchanged (Fig 5B). Oxidative phosphorylation supported by complex I (P_I+β_), complex II (P_II+β_) and both combined (P_I+II+β_) were all increased by sustained hypoxia, by 40% (*p* ≤ 0.05), 42% (*p* ≤ 0.05) and 42% (*p* ≤ 0.05), respectively (Fig 5B). Neither short-term, nor sustained hypoxia altered mitochondrial coupling (OCE) (Fig 5C), but preference for FAO was lowered by 12% (*p* ≤ 0.05) after sustained but not short-term hypoxia in the soleus (Fig 5D).

### Regulation of metabolism in the soleus

**Fig 6:**
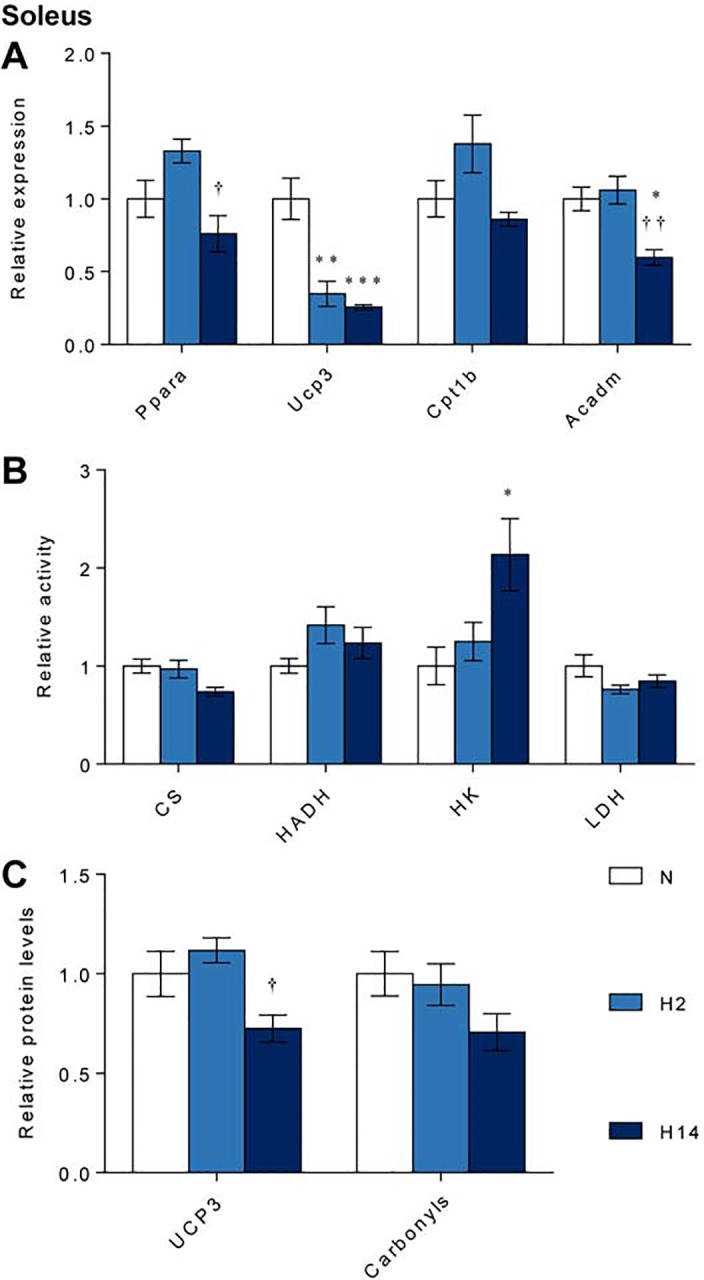
Regulation of metabolism in the soleus. mRNA levels of *Ppara* and its targets (A); activity of citrate synthase (CS), 3-hydroxyacyl dehydrogenase (HADH), hexokinase (HK) and lactate dehydrogenase (LDH) (B); and levels of UCP3 and protein carbonyls (C) after normoxia (N, white), short-term hypoxia (H2, light blue) or sustained hypoxia (H14, dark blue). * *p* ≤ 0.05, ** *p* ≤ 0.01 *vs*. normoxia; † *p* ≤ 0.05 *vs*. short-term hypoxia. Data represent mean ± SEM.

*Ppara* expression in the soleus did not differ from normoxic levels after either duration of hypoxic exposure (Fig 6A). *Ucp3* expression however, was 65% (p ≤ 0.01) and 74% (p ≤ 0.001) lower than normoxic controls following short-term and sustained hypoxia, respectively (Fig 6A). As in the heart, expression of *Acadm* fell by 40% (p ≤ 0.05) after sustained hypoxia, although *Cpt1b* was unchanged at both hypoxic timepoints (Fig 6A). There was a near-significant decrease in CS activity in the skeletal muscle following sustained hypoxic exposure (*p* = 0.056) (Fig 6B). HADH and LDH activity were unchanged, while HK activity increased 2.1-fold (*p* ≤ 0.05) with exposure to sustained hypoxia.

UCP3 protein levels did not differ significantly between normoxic controls and either hypoxic group (Fig 6C). Protein carbonyl levels were unchanged in soleus, suggesting that hypoxia-induced oxidative stress did not occur after short-term or sustained hypoxic exposure (Fig 6C).

## Discussion

The main finding of this study is that a substrate switch away from fatty acid oxidation towards glycolysis/pyruvate oxidation was observed in both the left ventricle and soleus following sustained but not short-term exposure to environmental hypoxia, with the ratio between oxidative phosphorylation supported by octanoyl carnitine *vs*. pyruvate decreased and hexokinase activity increased in both tissues. Expression of *Ppara* target genes were lowered following sustained hypoxic exposure, though expression of *Ppara* itself was unchanged. In addition, mass-specific respiratory capacity decreased in the left ventricle but not the soleus after sustained exposure to hypoxia. However, while citrate synthase-corrected respiration rates were unchanged in the left ventricle, these were increased in the soleus. A transient increase in oxidative stress was observed in the hypoxic left ventricle but not the hypoxic soleus. Finally, there was a marked decrease in *Ucp3* expression with both short-term and sustained hypoxia in both tissues, however, no change in UCP3 protein levels or mitochondrial coupling was observed in either tissue.

In this study, the metabolic effects of environmental hypoxia were compared in two tissues at two distinct timepoints. Since a loss of respiratory capacity could be due to a specific alteration of intra-mitochondrial function or a loss of mitochondrial volume density, respiration rates were corrected both to wet mass of muscle fibres, to indicate mass-specific respiration, and citrate synthase activity, to indicate respiration relative to TCA cycle activity. This novel method of normalising respiration rates to both parameters in tandem allowed us to discern qualitative changes in mitochondrial function and function of the mitochondrial network in its entirety. Citrate synthase activity has been shown to correlate with mitochondrial density in healthy, young men [29], though it is unknown whether this correlation is retained under hypoxic conditions. In addition, possible mechanisms to explain the changes in mitochondrial function were explored. Previous studies have considered multiple timepoints of exposure to hypoxia [15,17] and the tissue-specificity of the hypoxic response [30,31]. However, a consensus is yet to be reached on which tissues are susceptible to a substrate switch and/or a loss of mitochondrial mass following onset of hypoxia.

A possible limitation of the study is that only a 2 d normoxic control group was used for both 2 and 14 d hypoxic exposure, however we would not expect 12 further days of life under controlled conditions to induce measurable changes in metabolism, and a similar approach has been adopted by others [15]. Only the left ventricle of the heart and the soleus were used in this study, due to limitations of equipment availability, as fresh tissue was required for respirometry. A comparison of metabolic changes in the two ventricles, and between different skeletal muscles, would be of interest. The left ventricle was selected for this study as it is less susceptible to hypoxia-induced hypertrophy than the right ventricle, and hypertrophy would confound findings. The soleus was studied as it is a highly oxidative muscle, and thus a suitable tissue to investigate mitochondrial function. Moreover, in comparison with mixed fibre-type muscles, respirometry findings would not be confounded by selection of different fibre types when preparing tissue for respirometry.

Our findings suggest that in both left ventricle and soleus, metabolism is re-programmed in response to sustained exposure to hypoxia to increase the capacity for anaerobic metabolism and lower that for fatty acid oxidation, which is less O_2_-efficient than glycolysis/pyruvate oxidation [32]. The suppression of fatty acid oxidation relative to pyruvate oxidation appeared to be brought about by a decrease in transcriptional activity of *Ppara*, but not a downregulation of *Ppara* itself, which is most likely the result of a HIF-dependent decrease in PPARα DNA binding activity [9]. The increase in hexokinase activity in both tissues is also most likely a consequence of HIF signalling, as HIF-1α is known to increase expression of glycolytic enzymes [33].

There was no change in any measurement of mass-specific mitochondrial function in soleus. Unexpectedly, citrate synthase-corrected oxidative phosphorylation respiration rates (with the exception of that supported by octanoyl carnitine and glutamate) increased after sustained hypoxic exposure. Moreover, there was a near-significant decrease in citrate synthase activity (*p* = 0.056). Taken together, this suggests that mitochondrial oxidative phosphorylation capacity is relatively preserved in the hypoxic soleus in comparison with other mitochondrial pathways. This may indicate a specific loss of citrate synthase or of other TCA cycle enzymes, but since mass-specific mitochondrial function did not change it does not seem to indicate a wholesale loss of muscle mitochondrial content.

In contrast with soleus, the ratio between oxidative phosphorylation and citrate synthase activity remained constant in the left ventricle following hypoxic exposure, whereas mass-specific mitochondrial function decreased. Mitochondrial density is substantially higher in heart than in skeletal muscle. Under hypoxic conditions, mitochondrial ROS production increases through the stabilisation of ubisemiquinone when *P*_O2_ is low, resulting in more frequent partial reduction of O_2_ by ubisemiquinone to produce O_2_∙^-^ [1]. The lower oxygen consumption of skeletal muscle, particularly under resting conditions, may help to maintain mitochondrial *P*_O2_ in hypoxia, thereby minimising oxidative stress and maintaining mitochondrial function. In this study, we observed transient oxidative stress in the left ventricle, where mitochondrial function was compromised, but not in the soleus where it remained unchanged. Given a sufficiently severe hypoxic stimulus, skeletal muscle mitochondrial function has been shown to be attenuated in a manner that is reversed by the anti-oxidant, vitamin E [34,35], suggesting that ROS may play a key role in hypoxia-induced attenuation of mitochondrial function. ROS may control this effect through the stabilisation of HIF [36], which is known to upregulate BNIP3 thus resulting in mitochondrial degradation [7].

Interestingly, we found that expression of *Ucp3* was markedly decreased after both short-term and sustained exposure to hypoxia in both left ventricle and soleus despite a transient increase in oxidative stress in the left ventricle. This was perhaps surprising, as UCP3 expression has been shown to be enhanced by the presence of ROS in cell lines [37] and downregulation of other PPARα targets only occurred after sustained hypoxia. Moreover, in humans acclimatising to altitude, a possible increase in skeletal muscle UCP3 expression occurred with short-term exposure, with a decrease in UCP3 only with more prolonged exposure to extreme high altitude [21]. Despite this change in expression, however, no change in protein levels of UCP3, and thus no change in or oxidative phosphorylation coupling efficiency (a measure of mitochondrial coupling) was observed at either timepoint. Our data is therefore in agreement with a previous study [14], which showed decreased *Ucp3* expression at the RNA level with no change in mitochondrial coupling, although UCP3 protein levels were not reported.

Further work is required to elucidate the mechanisms which underlie the physiological responses to hypoxia described in this study. While oxidative phosphorylation increases relative to citrate synthase activity in the soleus of hypoxic rats, the mechanisms underlying this remain unknown. More specific investigation into expression and activity levels of enzymes in the TCA cycle and electron transport chain, perhaps using high-throughput metabolomics [38] may aid our understanding of this process. Furthermore, the mechanism by which *Ucp3* expression is downregulated earlier than other targets of PPARα remains to be elucidated.

## Conclusion

Environmental hypoxia for 14 days, but not 2 days, lowers mitochondrial respiratory function in rat left ventricle, whilst no such change occurs in the soleus. Qualitative changes in mitochondrial function are induced by hypoxia in both tissues, as a substrate switch away from fatty acid oxidation to glycolysis/pyruvate oxidation occurs following 14 days of hypoxia. *Ucp3* expression is decreased by both 2 and 14 days of hypoxic exposure, yet this is not reflected in a change in protein levels or mitochondrial coupling.

## Supporting Information

S1 Dataset Full dataset for manuscript. (XLSX)
